# Genomic and phenotypic comparison of two variants of multidrug-resistant *Salmonella enterica* serovar Heidelberg isolated during the 2015–2017 multi-state outbreak in cattle

**DOI:** 10.3389/fmicb.2023.1282832

**Published:** 2023-10-20

**Authors:** Selma Burciaga, Julian M. Trachsel, Donald Sockett, Nicole Aulik, Melissa S. Monson, Christopher L. Anderson, Shawn M. D. Bearson

**Affiliations:** ^1^United States Department of Agriculture, Agriculture Research Services, National Animal Disease Center, Ames, IA, United States; ^2^Oak Ridge Institute for Science and Education (ORISE), ARS Research Participation Program, Oak Ridge, TN, United States; ^3^Wisconsin Veterinary Diagnostic Laboratory, University of Wisconsin, Madison, WI, United States

**Keywords:** *Salmonella*, outbreak, pathogenicity, virulence, dairy beef calves, Heidelberg

## Abstract

*Salmonella enterica* subspecies *enterica* serovar Heidelberg (*Salmonella* Heidelberg) has caused several multistate foodborne outbreaks in the United States, largely associated with the consumption of poultry. However, a 2015–2017 multidrug-resistant (MDR) *Salmonella* Heidelberg outbreak was linked to contact with dairy beef calves. Traceback investigations revealed calves infected with outbreak strains of *Salmonella* Heidelberg exhibited symptoms of disease frequently followed by death from septicemia. To investigate virulence characteristics of *Salmonella* Heidelberg as a pathogen in bovine, two variants with distinct pulse-field gel electrophoresis (PFGE) patterns that differed in morbidity and mortality during the multistate outbreak were genotypically and phenotypically characterized and compared. Strain SX 245 with PFGE pattern JF6X01.0523 was identified as a dominant and highly pathogenic variant causing high morbidity and mortality in affected calves, whereas strain SX 244 with PFGE pattern JF6X01.0590 was classified as a low pathogenic variant causing less morbidity and mortality. Comparison of whole-genome sequences determined that SX 245 lacked ~200 genes present in SX 244, including genes associated with the IncI1 plasmid and phages; SX 244 lacked eight genes present in SX 245 including a second YdiV Anti-FlhC(2)FlhD(4) factor, a lysin motif domain containing protein, and a pentapeptide repeat protein. RNA-sequencing revealed fimbriae-related, flagella-related, and chemotaxis genes had increased expression in SX 245 compared to SX 244. Furthermore, SX 245 displayed higher invasion of human and bovine epithelial cells than SX 244. These data suggest that the presence and up-regulation of genes involved in type 1 fimbriae production, flagellar regulation and biogenesis, and chemotaxis may play a role in the increased pathogenicity and host range expansion of the *Salmonella* Heidelberg isolates involved in the bovine-related outbreak.

## 1. Introduction

Non-typhoidal *Salmonella* is one of the top five foodborne pathogens and one of the leading causes of bacterial foodborne illness in humans in the United States (U.S.) and worldwide (Scallan et al., 2013; Havelaar et al., 2015). *Salmonella enterica* subspecies *enterica* serovar Heidelberg (*Salmonella* Heidelberg) is primarily isolated from poultry, although it can colonize other hosts and cause salmonellosis in humans (CDC, 2013; Clothier and Byrne, 2016). Like other *Salmonella* serovars, *Salmonella* Heidelberg is typically transmitted from animals to humans *via* contaminated food sources. Also similar to other *Salmonella* serovars, *Salmonella* Heidelberg usually colonizes animals without resulting in clinical disease, but frequently causes gastroenteritis in humans. Over the last decade, six multistate outbreaks of *Salmonella* Heidelberg occurred in the U.S., and five of the outbreaks were associated with consumption of contaminated chicken-or turkey-related products (Antony et al., 2018). The most recent *Salmonella* Heidelberg outbreak was linked to direct contact with dairy beef calves (defined as an intact male dairy calf) from January 2015 through November 2017. Fifty-six people reported infections with multidrug-resistant (MDR) *Salmonella* Heidelberg across 15 states, the majority from Wisconsin. During the course of the outbreak, 54 people were interviewed with 34 (63%) reporting contact with dairy beef calves which were later shown to be infected with MDR *Salmonella* Heidelberg (CDC, 2017). Some of the calves infected with outbreak strains of MDR *Salmonella* Heidelberg displayed signs of disease such as diarrhea and fever, frequently followed by death from generalized bacteremia/septicemia (Sockett et al., 2017). Pulse-field gel electrophoresis (PFGE) and whole-genome sequencing (WGS) conducted on outbreak-associated isolates from sick calves revealed that human-and bovine-origin *Salmonella* Heidelberg isolates were closely related (Nichols et al., 2022). Furthermore, two main variants of *Salmonella* Heidelberg were identified in the cattle population by PFGE (Nichols et al., 2022). One of the variants was dominant and highly pathogenic causing 25–65% of the deaths in dairy beef calves, while the other variant was less pathogenic causing considerably fewer deaths (Sockett et al., 2017; Nichols et al., 2022).

In the current study, two outbreak isolates with varying virulence in calves were genotypically and phenotypically compared to identify factors that may have contributed to the emergence and pathogenic variation of *Salmonella* Heidelberg in the bovine species. Gene content was compared based on whole genome sequencing (WGS), differences in gene expression patterns were revealed through RNA-seq between the isolates when grown in culture, and phenotypic comparisons assessed their invasion of human and bovine epithelial cells.

## 2. Materials and methods

### 2.1. *Salmonella* isolates and growth conditions

*Salmonella* was isolated and identified from bovine cases submitted to the Wisconsin Veterinary Diagnostic Laboratory (WVDL) at the University of Wisconsin-Madison during the 2015–2017 multistate outbreak as previously described by Nichols et al. (2022). Two bovine-origin *Salmonella* Heidelberg isolates were received from the WVDL and referred to hereafter as SX 244 and SX 245; WVDL determined the PFGE patterns for SX 244 (JF6X01.0590) and SX245 (JF6X01.0523). Bacteria were streaked from the frozen glycerol stock solution onto Luria-Bertani (LB; Lennox) agar (Invitrogen, Waltham, MA) and incubated at 37°C overnight. Individual colonies were selected and inoculated into 3 mL of LB broth (Invitrogen) at 37°C overnight with shaking for further analysis.

### 2.2. DNA isolation, whole-genome sequencing, and analysis

Overnight *Salmonella* cultures were centrifuged for 20 min at 3000 × g. Supernatants were removed and cell pellets resuspended in 400 μL of phosphate buffered saline. DNA isolation was performed on 100 μL of the resuspension using the High Pure PCR Template Preparation Kit (Roche Applied Science, Indianapolis, IN) per manufacturer’s instructions. The quality and quantity of DNA were measured on the Qubit 4 Fluorometer using the Qubit™ dsDNA Broad Range Assay Kit (Invitrogen). WGS libraries were generated using the Nextera DNA Flex Library Prep and indices kits (Illumina, San Diego, CA) and sequenced using the MiSeq reagent kit v3 (600-cycle) yielding 2 × 300-bp paired-end reads on the Illumina MiSeq platform (Illumina).

FastQC v0.11.6 was used to evaluate the quality of raw Illumina reads and determine the total number of reads (Andrews and Fast, 2014). Sequencing adapters and artifacts were removed from the short reads using BBtools v38.30 (Bushnell, 2018). Genome assemblies were generated using the *de novo* assembler SPAdes v3.11.1 (Bankevich et al., 2012), and the quality of the assemblies were assessed using QUAST v4.6.3 (Gurevich et al., 2013). SX 244 and SX 245 genomes were annotated with prokka v1.14.6 (Seemann, 2014) using proteins from *Salmonella enterica* serovar Heidelberg strain SL476 (accession GCA_000020705.1) as a first priority for the annotation. Gene ontology (GO) terms were assigned to genes in the SX 244 and SX 245 genomes with interproscan v5.35–74.0 (Jones et al., 2014), and PPanGGOLiN v1.0.1 (Gautreau et al., 2020) was used to identify genes that were shared (core genes) or unique to each genome.

### 2.3. RNA extraction, RNA sequencing, and transcriptional analysis

Overnight *Salmonella* cultures were diluted 1:200 in LB broth and grown to OD_600_ = 0.3 (early log phase growth) *via* shaking at 37°C. An 0.5 mL aliquot of each culture was placed in RNAprotect™ Bacteria Reagent (Qiagen, Germantown, MD) and processed per manufacturer’s instructions to provide immediate stabilization of RNA. Cultures and RNA isolations were repeated three times per isolate (three biological replicates). RNA was extracted using the RNeasy Mini Kit (Qiagen), followed by treatment with TURBO™ DNase (Ambion, Austin, TX, USA) to remove genomic DNA. A 2100 Bioanalyzer (Agilent Technologies, Santa Clara, CA) and Agilent RNA 6000 Nano kit (Agilent Technologies) were used to evaluate the quality of total RNA. Bacterial ribosomal RNA (rRNA) sequences were depleted using the Ribo-Zero Plus rRNA Depletion Kit (Illumina), and the quality of the rRNA depleted RNA was assessed using the 2100 Bioanalyzer. RNA libraries were constructed using the NEBNext^®^ Ultra™ II Directional RNA Library Prep Kit (New England BioLabs^®^, Ipswich, MA) and sequenced at the Iowa State University DNA Facility on an Illumina Hiseq 3000 (150 cycles, single-end reads; Illumina).

Quality of raw RNA sequencing data was assessed using FastQC v0.11.6. BBtools v38.30 was used to remove sequencing adapters and artifacts and to quality trim (average quality scores <10) the raw Illumina reads. RNA-seq reads of SX 244 and SX 245 were aligned to the genome sequence of SX 244 using BBtools v38.30 with default parameters, and read counts (the number of reads that aligned to a specific gene) were quantified using HTseq v0.11.0 (nonunique reads mapped to all) (Anders et al., 2015). Read counts were normalized and gene expression compared (by Wald test) between the two strains using DESeq2 v.1.34.0 (Love et al., 2014); log_2_ fold change (Log_2_FC) shrinkage was performed using apeglm v1.16.0 (Zhu et al., 2019). Principal component analysis (PCA) was performed to ascertain expression outliers based on variance stabilized gene expression counts for the top 200 most variable genes using DESeq2 v1.34.0 and pcaExplorer v2.27.1 (Marini and Binder, 2019). Final *p*-value for differential gene expression were adjusted with a Benjamini–Hochberg procedure (false discovery rate; FDR), with an FDR adjusted *p*-value <0.05 and |Log_2_FC| ≥ 0.50 considered as significant. Based on the GO terms assigned by interproscan v5.35–74.0, a GO term enrichment analysis (Ashburner et al., 2000) was conducted to predict functional consequences of the differentially expressed genes using a Fisher’s exact test in topGO v2.46.0 (Alexa and Rahnenfuhrer, 2021). The topGO analysis (“elimination” algorithm with a minimum node size of 5 genes) was conducted for all three major GO aspects: “biological process,” “molecular function,” and “cellular component” and any term with a *p*-value <0.05 was considered significantly enriched.

### 2.4. Invasion cell culture assays

For each biological experiment, an overnight culture was diluted 1:100 in fresh LB broth and grown with shaking for 1.5 h at 37°C to early-log phase (OD_600_ = 0.3) for the invasion assays. The human epithelial-like tumor cell line HEp-2 (ATCC: CCL-23) was grown and maintained in Gibco RPMI 1640 medium (Thermo Fisher Scientific, Waltham, MA) with Gibco 10% heat-inactivated fetal bovine serum (FBS) in an atmosphere of 5% CO_2_ at 37°C. Madin-Darby bovine kidney epithelial cells (MDBK; ATCC 6071) were grown and maintained in Gibco MEM (Thermo Fisher Scientific) supplemented with Gibco 10% heat-inactivated FBS, Gibco Antibiotic-Antimycotic (Anti:Anti), and Gibco L-glutamine.

For the invasion assays, HEp-2 and MDBK cells were seeded in 24-well cell culture plates (BD Falcon, BD Biosciences, San Jose, CA) at 1.3 × 10^5^ and 2 × 10^5^ cells, respectively, and incubated overnight at 37°C with 5% CO_2_ until >95% confluent. Invasion assays were performed with three technical replicates for each biological replicate using a gentamicin protection assay in HEp-2 and MDBK cells with a multiplicity of infection (MOI) ratio of 50:1 as previously described (Elsinghorst, 1994). Three biological replicates were performed for HEp-2 invasion assays and five for MDBK invasion assays. Percent invasion was calculated by dividing colony forming units (CFU) of bacteria recovered by CFU of bacteria added to the cells and multiplying by 100. The significant differences between SX 244 and SX 245 invasion were determined by unpaired Student’s *t-test* using GraphPad Prism 9 (Birhanu et al., 2018; Mechesso et al., 2021). *p*-values less than 0.05 were considered significant.

### 2.5. Antimicrobial susceptibility testing

Antimicrobial susceptibility (AST) of the two *Salmonella* Heidelberg strains was assessed using the Sensititre™ National Antimicrobial Resistance Monitoring System (NARMS) Gram Negative CMV4AGNF AST plate by the National Veterinary Services Laboratories. The CMV4AGNF plate contained 14 antimicrobials in different antibiotic classes including aminoglycosides (gentamicin and streptomycin), penicillin (ampicillin), beta-lactam combinations (amoxicillin/clavulanic acid), cephalosporins (ceftriaxone and cefoxitin), carbapenems (meropenem), macrolides (azithromycin), quinolones/fluoroquinolones (ciprofloxacin and nalidixic acid), phenicol (chloramphenicol), folate pathway antagonists (sulfisoxazole and trimethoprim/sulphamethoxazole), and tetracyclines (tetracycline). The strains were classified as susceptible, intermediate, or resistant as defined by the Clinical and Laboratory Standards Institute (CLSI, 2023), when available. Otherwise, NARMS consensus breakpoints were used. Multidrug resistance (MDR) was defined as resistant to three or more antimicrobial classes and decreased susceptibility to ciprofloxacin (DSC, MIC ≥0.12 μg/mL) was defined by NARMS.

## 3. Results and discussion

### 3.1. *Salmonella* Heidelberg genomes

During the 2015–2017 multistate outbreak of MDR *Salmonella* Heidelberg linked to dairy beef calf exposure, SX 245 was identified as a dominant and highly pathogenic variant causing 25–65% of the deaths in dairy beef calves, while SX 244 was a less pathogenic variant causing considerably fewer deaths (Sockett et al., 2017; Nichols et al., 2022). To identify potential pathogenic characteristics of bovine-origin MDR *Salmonella* Heidelberg strains from this outbreak, genome sequencing and analysis of the two *Salmonella* Heidelberg strains were performed and identified 4,960 features in SX 244 and 4,761 in SX 245 (Table 1). After filtering to exclude non-protein-coding sequences, genomic comparisons revealed 4,670 protein-coding genes shared between both strains. SX 245 lacked 204 genes that were present in SX 244 (Supplementary Table S1). Approximately half (52%) of the 204 genes were hypothetical proteins with unknown function. The remaining genes included several genes associated with the IncI1 plasmid such as conjugal transfer proteins and plasmid thin pilus genes, as well as genes associated with bacteriophages involved in recombination and replication. In contrast, SX 244 lacked 8 genes that were present in SX 245, five of which were unknown hypothetical proteins (Supplementary Table S1). The remaining three genes encode a YdiV Anti-FlhC(2)FlhD(4) factor (*ydiV*), a peptidoglycan DD-metalloendopeptidase family protein with a lysin motif (LysM) domain, and a pentapeptide repeat protein. The genome of SX 244 contained a single *ydiV* gene, while SX 245 contained two copies of this gene. YdiV suppresses the activity of FlhD_4_C_2_, a master regulator of flagellar gene expression, by binding to the FlhD region of the complex further inhibiting transcription of the class II gene *fliA* required for advancing flagella biosynthesis (Wada et al., 2011). Repression of flagellar genes can be beneficial to *Salmonella* during its pathogenesis. For example, YdiV represses flagellar genes in response to nutritional cues, such as poor nutrient conditions inside macrophages (Stewart et al., 2011; Wada et al., 2011). Additionally, YdiV represses flagellar genes in systemic tissues, which protects *Salmonella* from caspase-1-mediated bacterial clearance (Lara-Tejero et al., 2006; Miao et al., 2006; Stewart et al., 2011). The regulation of *Salmonella* flagellar expression reflects the importance of reducing flagella in specific environments for survival. The LysM domain is associated with peptidoglycan binding and is found in various enzymes involved in bacterial cell wall degradation (Joris et al., 1992), comparable to those in peptidoglycan hydrolases (Buist et al., 2008). LysM is reported to enhance survival in macrophages and is needed for systemic infection and pathogenicity in *Salmonella* Enteritidis (Silva et al., 2012).

**Table 1:** Genomic content of *Salmonella* Heidelberg SX 244 and SX 245 strains.

Feature type		SX 244 (Low pathogenicity)	SX 245 (High pathogenicity)
Protein-coding genes (CDS)	Shared	4,670	4,670
	Unique	204	8
	Total	4,874	4,678
Ribosomal RNAs (rRNAs)		9	9
Transfer messenger RNAs (tmRNAs)		1	1
Transfer RNAs (tRNAs)		76	73
Total		4,960	4,761

The presence of a second *ydiV* gene and an additional gene encoding an enzyme with a LysM domain could potentially provide SX 245 with a fitness advantage over SX 244 in the host. Furthermore, the deletion of over 200 genes may benefit SX 245 because maintenance of superfluous genes can be a liability (Ehrenberg and Kurland, 1984; Waters et al., 2022). Deletion of these genes could enhance *Salmonella* fitness as more resources are available for allocation to other rate-limiting processes (Koskiniemi et al., 2012), suggesting the selection and expression of *Salmonella* Heidelberg with a reduced genome may be a driver in the evolution of adaptation and virulence to the bovine host.

### 3.2. Bacterial transcriptome

**Figure 1:**
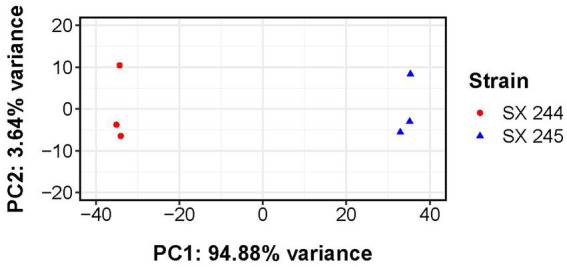
Principal component analysis of transcriptomes from *Salmonella* Heidelberg strains SX 244 and SX 245. Variance-stabilized read counts for the 200 most variable genes were used for this analysis. Principal component 1 (PC1) and principal component 2 (PC2) are shown for each strain (indicated by color and shape).

**Figure 2:**
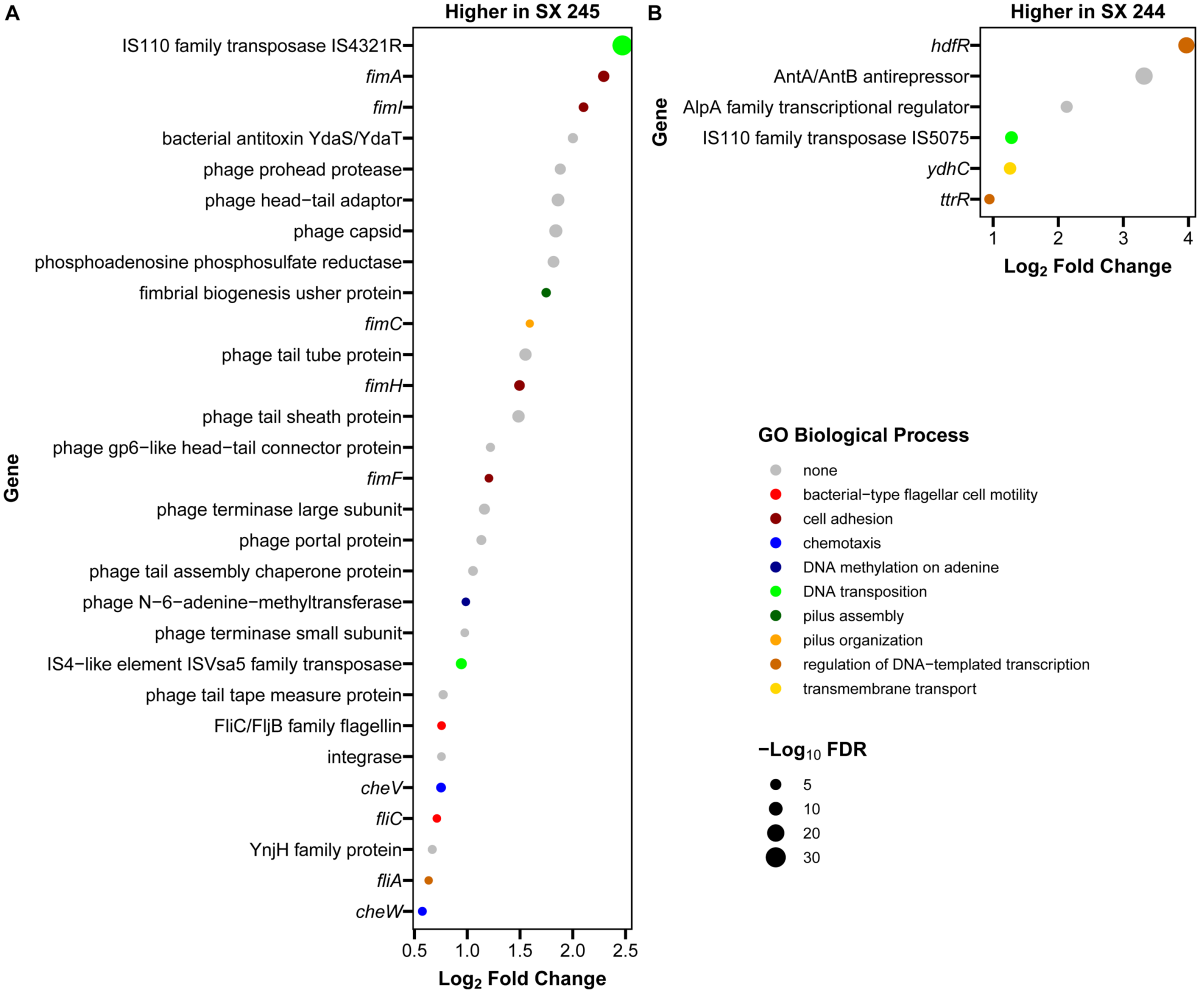
Annotated genes with differential expression between *Salmonella* Heidelberg strains SX 244 and SX 245. Log_2_ fold changes were filtered for significant differential expression (|log_2_ fold change| ≥ 0.5, FDR < 0.05,) and for genes that were annotatable (prokka and interproscan) and present in both the SX 244 and SX 245 genomes. Genes with higher expression in SX 245 **(A)** and in SX 244 **(B)** are shown, along with functions predicted by associated GO Biological Process terms.

RNA-Seq was performed to compare gene expression patterns between the high and low pathogenic *Salmonella* Heidelberg strains in broth culture. Mapping reads to SX 244 as the reference genome provided evidence for expression of 99% of these genes in SX 244 (4,948 genes on average) and 97% of these genes in SX 245 (4,796 genes on average). PCA on the 200 genes with the highest variability between datasets revealed that strain is the main factor driving these transcriptomes, accounting for nearly 95% of the variation in expression (Figure 1). Differential gene expression (|Log_2_FC ≥ 0.50|, FDR < 0.05) was observed for 246 genes; however, 194 were unique genes that were not present in SX 245 and another five were transfer RNAs (tRNAs), which were not compared across genomes (Supplementary Table S2). Of the 47 differentially expressed genes that were in common between both genomes, 35 genes (74%) had higher expression in SX 245 than SX 244. Twenty-nine of the genes upregulated in the highly pathogenic strain SX 245 were annotatable by prokka and/or interproscan, identifying virulence genes such as fimbriae-related genes (*fimA, fimI, fimC, fimH, fimF*, and a fimbrial biogenesis usher protein), flagella-related genes (*fliA, fliC*, and another flagellin FliC/FljB family member), and genes involved in chemotaxis (*cheW, cheV*) (Figure 2A; Supplementary Table S2). GO enrichment analysis confirmed that genes with higher expression in SX 245 had overrepresented biological functions such as “chemotaxis,” “DNA transposition,” “bacterial-type flagellar cell motility,” and “cell projection organization” (Figure 2A; Supplementary Table S3). Among the genes upregulated in SX 245, eleven genes that lacked associations with GO Biological Process terms (classified as “none” (gray circles) in Figure 2A) as well as one DNA methyltransferase gene appeared to be associated with bacteriophage composition and function based on homology identified with interproscan (Supplementary Table S2). Six genes upregulated in SX 245 were hypothetical proteins of unknown function. Twelve differentially expressed genes (shared by both genomes) had higher expression in SX 244 compared to SX 245, of which six were annotatable and four had GO term associations (Figure 2B; Supplementary Table S2).

The *fim* genes exhibited some of the greatest increases in expression in SX 245 compared to SX 244 (|Log2FC ≥ 0.5|, FDR < 0.05). *FimA*, *fimI*, *fimC*, *fimH*, and *fimF* are structural genes necessary for type 1 fimbriae (T1F) production and are expressed in a single operon under the control of the *fimA* promoter region (Purcell et al., 1987; Rossolini et al., 1993). *Salmonella* contains several fimbriae across their surface which play a vital role in adhesion and invasion to establish colonization as well as maintain infection (van der Velden et al., 1998). T1F are important for *Salmonella* entry into epithelial cells and intestinal colonization of several hosts (Darekar and Duguid, 1972; Duguid et al., 1976; Ernst et al., 1990; Dibb-Fuller and Woodward, 2000; Wilson et al., 2000). Prior studies investigating the role of T1F in *Salmonella* pathogenesis in animal models show that *Salmonella* Enteritidis expressing T1F are more infectious and virulent than non-fimbriated strains in mouse infection models (Darekar and Duguid, 1972; Duguid et al., 1976). Similarly, wildtype *Salmonella* Enteritidis colonizes the spleen, liver, and ceca of 1-day old chicks in significantly greater loads than a mutant strain unable to express T1F (Dibb-Fuller and Woodward, 2000). Wilson et al. (2000) demonstrated that *Salmonella* serovars Pullorum and Gallinarum expressing Typhimurium T1F display an increased ability to adhere (10-to 20-fold) and invade (20-to 60-fold) the human epithelial HEp-2 cell line. In addition to the highly expressed *fim* operon observed in SX 245, other fimbrial operons were identified in the genomes of SX 244 and SX 245. Genomic comparisons of 617 publicly available *Salmonella* Heidelberg isolates from the National Center for Biotechnology and 17 *Salmonella* Heidelberg isolates from cases submitted to the Animal Disease Research and Diagnostic Laboratory, South Dakota State University and WVDL identified the *saf* operon as the defining feature of outbreak-associated human/bovine isolates (Antony et al., 2018). *Salmonella* atypical fimbriae (Saf) is also important for pathogenesis, particularly in *Salmonella* associated with human disease (Folkesson et al., 1999; Sheikh et al., 2010; Bhuiyan et al., 2014). Therefore, the up-regulation of the *fim* operon along with the presence of the *saf* operon may have contributed to the disease severity of these outbreak-associated *Salmonella* Heidelberg strains in bovine.

Flagella are an important virulence factor of *Salmonella* that allow for motility and chemotaxis to reach sites of infection and evade host, immune responses (Josenhans and Suerbaum, 2002; Duan et al., 2013). Flagella are also required for efficient replication and colonization in the lumen of an inflamed intestine (Stecher et al., 2004, 2008). Along with an unspecified flagellin family member, the flagella-related genes *fliA* and *fliC* had increased expression in SX 245, and these two genes encode the flagella-specific sigma factor (σ^28^) and phase 1 flagellin (FliC) in *Salmonella*, respectively. Previous studies indicate that FliA positively regulates all class III promoters involved in flagellar biosynthesis controlling the expression of genes responsible for the major subunits of flagella (*fliC*, *fljB*), motility (*motAB*) and chemotaxis (*cheAW*) (Ohnishi et al., 1990; Chilcott and Hughes, 2000; Erhardt et al., 2014). *Salmonella* expressing FliC-flagella have an advantage in motility dependent invasion and target-site selection during swimming in gut colonization in murine gastroenteritis infection models (Bogomolnaya et al., 2014; Horstmann et al., 2017). *Salmonella* has an arsenal of virulence mechanisms used to establish infection; higher expression of these virulence genes may play a role in the pathogenicity of outbreak-associated *Salmonella* Heidelberg in dairy beef calves.

### 3.3. Invasion of epithelial cells

**Figure 3:**
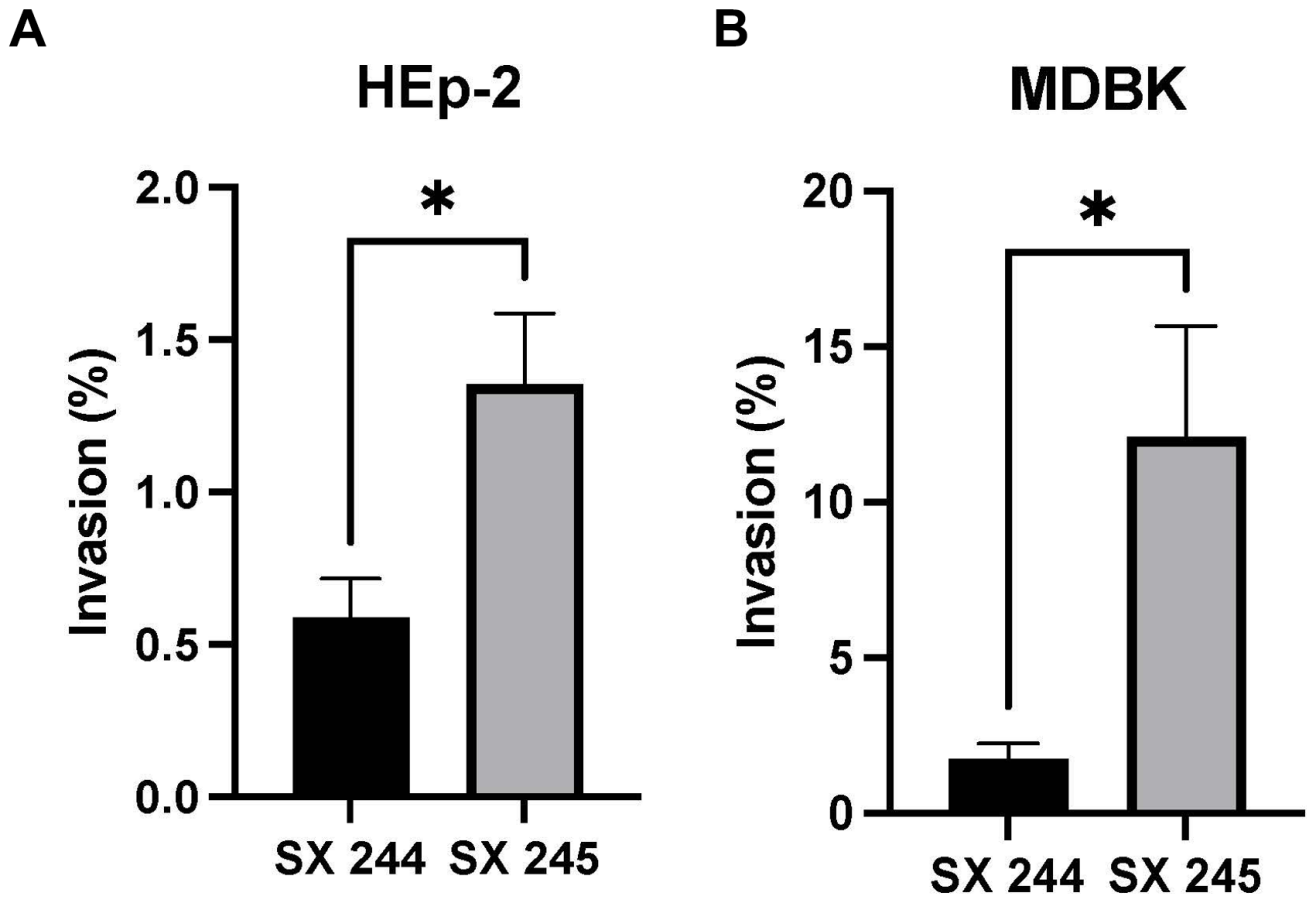
Percent invasion of *Salmonella* Heidelberg strains SX 244 and SX 245. Invasion assays were performed on SX 244 and SX 245 in **(A)** HEp-2 human epithelial cells (*p*-value = 0.0446) and **(B)** MDBK bovine epithelial cells (*p*-value = 0.0197).

*Salmonella* invasion of epithelial cells (a first line of defense against intestinal pathogens) is an important phenotype associated with virulence (Wallis Timothy and Barrow, 2005; Gal-Mor et al., 2014; Cheng et al., 2019). Because differential expression of genes involved in invasion was detected, invasion assays were conducted to compare the invasiveness of the high (SX 245) and low (SX 244) pathogenic *Salmonella* Heidelberg strains in human (HEp-2) and bovine (MDBK) epithelial cells. SX 245 (1.35%) had a significantly higher invasion rate (>2-fold) than SX 244 (0.58%) of human epithelial cells (Figure 3A; *p*-value <0.05). The percent invasion of MDBK by SX 245 (12.12%) was also significantly higher (>7-fold) compared to SX 244 (1.73%) (Figure 3B; *p*-value <0.05). The results of this study indicate SX 245 has greater invasive ability than SX 244 in bovine and human epithelial cells, which may be influenced by the increased expression of genes involved in T1F production, flagella regulation and biogenesis, and chemotaxis in SX 245.

While extensive cell invasion studies have been performed for *Salmonella* Typhimurium and *Salmonella* Enteritidis, limited studies are available for other serovars such as *Salmonella* Heidelberg (Ernst et al., 1990; Horiuchi et al., 1992; Bäumler et al., 1996; Brackelsberg et al., 1997; Wilson et al., 2000; Ledeboer et al., 2006; Kolenda et al., 2018; Campioni et al., 2021). *Salmonella* Typhimurium and *Salmonella* Braenderup with T1F are known to adhere and invade human cervical cancer (HeLa) cells with greater numbers than non-fimbriated strains (Horiuchi et al., 1992; Bäumler et al., 1996). Hancox et al. (1997) also observed greater adhesion to HEp-2 and HeLa cells of wildtype *Salmonella* Typhimurium than isogenic *fimH* mutants lacking T1F. Along with the current study, these results suggest T1F plays a role in enhancing cellular invasion of *Salmonella*. In contrast, other investigators used *fimH* mutants to report that T1F does not contribute to *Salmonella* Typhimurium adhesion or invasion of HEp-2 cells (Bäumler et al., 1996; Kolenda et al., 2018). These conflicting results may be due to the variability in experimental design such as differences in MOI, incubation times or the use of different *Salmonella* serovars or strains.

Two studies of *Salmonella* invasion in MDBK epithelial cells compared bovine-adapted *Salmonella* Dublin to host-generalist *Salmonella* Typhimurium and poultry-adapted *Salmonella* Enteritidis (Brackelsberg et al., 1997; Campioni et al., 2021). One reported that *Salmonella* Dublin has greater capability for invasion of MDBK cells than *Salmonella* Typhimurium, which may explain the association of *Salmonella* Dublin with severe forms of salmonellosis in cattle (Brackelsberg et al., 1997). The second study described similar MDBK invasion rates for host-adapted serovars *Salmonella* Enteritidis and *Salmonella* Dublin (Campioni et al., 2021). Invasion rates of these serovars are higher than the *Salmonella* Heidelberg strains in this study, at 75 and 73%, respectively. Brackelsberg et al. (1997) and Campioni et al. (2021) additionally compared strains within the same serovar and described varying rates of invasion of MDBK cells, which is congruent with other studies reporting *Salmonella* invasion being strain dependent (Betancor et al., 2009; Shah et al., 2011). Similarly, this study describes two *Salmonella* Heidelberg strains from the same outbreak with significantly different invasion rates for both MDBK and HEp-2 epithelial cells.

In summary, *Salmonella* can be transmitted from animals to humans directly through contact or indirectly through the food chain, resulting in zoonotic disease (Marshall and Levy, 2011). Thus, *Salmonella* is an animal and human health concern. *Salmonella* Heidelberg is primarily a poultry-associated serovar., linked to human illness *via* consumption of contaminated poultry products (Antony et al., 2018). However, this 2015–2017 multistate outbreak was unique because the *Salmonella* Heidelberg isolates associated with human disease were also associated with septicemia in dairy beef calves, which frequently led to calf death (Sockett et al., 2017; Nichols et al., 2022). Food animals typically harbor *Salmonella* as commensals and are usually subclinical (Stevens et al., 2009; Silva et al., 2014); therefore, the reason for the increased pathogenicity and disease severity of cattle-associated *Salmonella* Heidelberg is of particular interest. Two dominant variants of *Salmonella* Heidelberg were isolated during the outbreak that were similar in their genotypic MDR pattern but differed in their PFGE patterns (Nichols et al., 2022). Because greater morbidity and mortality in calves was associated with one variant (SX 245) compared to the other (SX 244), the present study compared virulence-related characteristics of the *Salmonella* Heidelberg isolates to explore their contrasting disease severity. Highly pathogenic SX 245 had elevated expression of virulence genes and greater invasion of human and bovine epithelial cells, potentially supporting the enhanced severity of *Salmonella* Heidelberg infection in dairy beef calves and eventual salmonellosis in humans (Supplementary Table S4). Altogether, comparison of the two strains suggests that genes involved in fimbriae production and flagellar biosynthesis may contribute to the increased pathogenicity and ecological success of *Salmonella* Heidelberg in the bovine species.

## Data availability statement

The genomic and transcriptomic datasets generated for this study are publicly available. The data can be found through NCBI BioProject PRJNA999325: https://www.ncbi.nlm.nih.gov/bioproject/999325. The raw data supporting the conclusions of the invasion assays will be made available by the authors upon request.

## Ethics statement

Ethical approval was not required for the studies on humans and animals in accordance with the local legislation and institutional requirements because only commercially available established cell lines were used.

## Author contributions

SB: Data curation, Formal analysis, Investigation, Methodology, Project administration, Software, Validation, Visualization, Writing – original draft, Writing – review & editing. JT: Data curation, Formal analysis, Investigation, Methodology, Software, Supervision, Validation, Writing – review & editing. DS: Conceptualization, Resources, Writing – review & editing. NA: Conceptualization, Resources, Writing – review & editing. MM: Data curation, Formal analysis, Methodology, Software, Validation, Visualization, Writing – original draft, Writing – review & editing. CA: Data curation, Methodology, Writing – review & editing. SMDB: Conceptualization, Data curation, Formal analysis, Investigation, Methodology, Project administration, Resources, Software, Supervision, Validation, Visualization, Writing – original draft, Writing – review & editing.
