# Supplementary material for: Genomic and phenotypic comparison of two variants of multidrug-resistant Salmonella enterica serovar Heidelberg isolated during the 2015–2017 multi-state outbreak in cattle
Source: Front Microbiol. 2023 Oct 20;14:1282832. doi: 10.3389/fmicb.2023.1282832 (PMC10623430; doi:10.3389/fmicb.2023.1282832)
Supplement: Supplementary file 4 [file Table_4.DOCX]

Supplementary Material

**Supplementary Table S4.** Phenotypic and genotypic comparisons of SX 244 and SX 245.

|  | SX 244 | SX 245 |
| --- | --- | --- |
| Deaths in dairy beef calves | Low pathogenicity | High pathogenicity |
| Isolation | Intestine of calves | Intestine of calves |
| Serovar | Heidelberg | Heidelberg |
| PFGE pattern | JF6X01.0590 | JF6X01.0523 |
| Genotypic antimicrobial resistance pattern | *aadA1*, *aph(3')-Ia*, *bla*_CMY-2_, *dfrA34*, *qnrB*19, *fosA7*,  *str*A-*str*B, *sul*1, *sul*2, *tet*(A), *tet*(B), *tet*(O) | *aadA1*, *aph(3')-Ia*, *bla*_CMY-2_, *dfrA34*, *qnrB*19, *fosA7*,  *str*A-*str*B, *sul*1, *sul*2, *tet*(A), *tet*(B), *tet*(O) |
| Phenotypic antimicrobial resistance pattern | Amoxicillin/Clavulanic Acid, Ampicillin, Cefoxitin, Ceftriaxone, Chloramphenicol, Nalidixic Acid, Streptomycin, Tetracycline, Trimethoprim/Sulphamethoxazole | Amoxicillin/Clavulanic Acid, Ampicillin, Cefoxitin, Ceftriaxone, Nalidixic Acid, Streptomycin, Tetracycline, Trimethoprim/Sulphamethoxazole |
| Total number of genes | 4,960 | 4,761 |
| Total number of up-regulated differentially expressed genes | 12  (plus 199 unique genes and tRNAs in SX 244) | 35 |
| GO enrichment | - regulation of DNA-templated transcription - DNA transposition - transmembrane transport | - bacterial-type flagellar cell motility - cell adhesion - chemotaxis - regulation of DNA-templated transcription |
| Invasion of HEp-2 cells | 0.58% | 1.35% |
| Invasion of MDBK cells | 1.73% | 12.12% |
